# *KIR2DL2* Enhances Protective and Detrimental HLA Class I-Mediated Immunity in Chronic Viral Infection

**DOI:** 10.1371/journal.ppat.1002270

**Published:** 2011-10-13

**Authors:** Nafisa-Katrin Seich al Basatena, Aidan MacNamara, Alison M. Vine, Chloe L. Thio, Jacquie Astemborski, Koichiro Usuku, Mitsuhiro Osame, Gregory D. Kirk, Sharyne M. Donfield, James J. Goedert, Charles R.M. Bangham, Mary Carrington, Salim I. Khakoo, Becca Asquith

**Affiliations:** 1 Imperial College, London, United Kingdom; 2 Johns Hopkins University, Baltimore, Maryland, United States of America; 3 Kumamoto University, Kumamoto, Japan; 4 Kagoshima University, Kagoshima, Japan; 5 Rho, Chapel Hill, North Carolina, United States of America; 6 National Cancer Institute, Rockville, Maryland, United States of America; 7 Cancer and Inflammation Program, Laboratory of Experimental Immunology, SAIC-Frederick, Inc., NCI-Frederick, Frederick, Maryland, United States of America; 8 Ragon Institute of MGH, MIT and Harvard, Boston, Massachusetts, United States of America; NIH/NIAID, United States of America

## Abstract

Killer cell immunoglobulin-like receptors (KIRs) influence both innate and adaptive immunity. But while the role of KIRs in NK-mediated innate immunity is well-documented, the impact of KIRs on the T cell response in human disease is not known. Here we test the hypothesis that an individual's KIR genotype affects the efficiency of their HLA class I-mediated antiviral immune response and the outcome of viral infection. We show that, in two unrelated viral infections, hepatitis C virus and human T lymphotropic virus type 1, possession of the *KIR2DL2* gene enhanced both protective and detrimental HLA class I-restricted anti-viral immunity. These results reveal a novel role for inhibitory KIRs. We conclude that inhibitory KIRs, in synergy with T cells, are a major determinant of the outcome of persistent viral infection.

## Author Summary

Hepatitis C virus (HCV) and human T-cell leukemia virus (HTLV-I) infect millions of people worldwide. Some HCV-infected individuals spontaneously clear the virus and many HTLV-1-infected people remain asymptomatic; however, in both cases the infection can lead to serious illness such as cancer. The factors which determine outcome are still elusive. We have found that a gene that encodes a receptor (KIR2DL2) enhances both protective and detrimental HLA class I-mediated immunity to HCV and HTLV-1. Strikingly, although KIRs are primarily associated with innate immunity, our observations suggest that they also have a major impact on the efficiency of the adaptive immune response. This work helps to explain why one individual infected with a virus remains healthy but another, infected with the same virus develops disease; it also helps to explain why particular HLA class I molecules do not always protect or cause susceptibility as expected. Interestingly, the impact of the KIR is entirely context dependent: if an HLA class I molecule is protective then protection is enhanced, but in the context of a detrimental HLA then susceptibility is enhanced. This study reveals a novel role for inhibitory KIRs in adaptive immunity.

## Introduction

Killer cell immunoglobulin-like receptors (KIRs) are a family of transmembrane proteins that are expressed on natural killer (NK) cells and subsets of T cells [1]–[3]. They bind HLA class I molecules and have activatory and inhibitory isoforms [4]. KIRs contribute directly and indirectly to antiviral immunity. Directly, KIRs on NK cells sense the loss of HLA class I molecules from the cell surface and trigger NK-mediated cytolysis. Indirectly, NK cells regulate adaptive immunity via crosstalk with dendritic cells and by the production of chemokines and cytokines [5]–[7].

HLA class I molecules can be grouped into allotypes with similar KIR binding properties [8]. For example, KIR2DL2 binds group C1 HLA-C molecules which have asparagine at residue 80, and, with a weaker affinity, group C2 molecules which have a lysine at position 80 [9].

Early research on KIRs investigated NK-mediated protection by studying disease associations with KIRs in the context of their HLA class I ligands [10]–[11]. There is now compelling evidence that KIRs also regulate adaptive immunity [5]–[7], but it is not known whether this has a significant impact on the response to infection in vivo. Differences between human KIRs and their mouse functional homologues (the Ly49 receptors) and the paucity of KIR allele-specific antibodies have hindered work on the role of KIRs in controlling adaptive immune responses. Here we used immunogenetics to investigate whether KIR genotype modulates HLA-mediated anti-viral protection in vivo. We focussed on HLA class I alleles which have previously been associated with disease outcome and investigated whether these effects were altered by the KIR background. We studied 4 well-documented HLA class I allele-disease associations in two viral infections: human T lymphotropic virus type 1 (HTLV-1) and hepatitis C virus (HCV).

HTLV-1 is a persistent retrovirus that infects 10–20 million people worldwide. Most infected individuals remain lifelong asymptomatic carriers (ACs). However, approximately 10% of infected individuals develop associated diseases including HTLV-1-associated myelopathy/tropical spastic paraparesis (HAM/TSP), an inflammatory disease of the central nervous system that results in progressive paralysis. It is poorly understood why some individuals remain asymptomatic whereas others develop disease, but one strong correlate of disease is the proviral load, which is significantly higher in HAM/TSP patients than in ACs [12]. We have previously shown that HLA-A*02 and C*08 are each associated with both a reduced risk of HAM/TSP and a reduced proviral load in ACs and that HLA-B*54 is associated with an increased prevalence of HAM/TSP and an increased proviral load in HAM/TSP patients [13]–[14].

HCV is among the most widespread viral infections, with 170 million infected people worldwide. As in HTLV-1 infection, the outcome of HCV infection is heterogeneous: the virus persists in approximately 70% of infected individuals while the rest clear the infection spontaneously. Chronic HCV infection can cause serious liver damage including cirrhosis and hepatocellular carcinoma [15]. The origins of this heterogeneity are not completely understood but several genetic determinants have been identified, including HLA-B*57 which is associated with spontaneous clearance in several cohorts [16]–[19].

The aim of this study was to test the hypothesis that KIR genotype determines the efficiency of HLA class I-mediated anti-viral immunity. We tested this hypothesis for 4 HLA class I associations: HLA-C*08, A*02 and B*54 in HTLV-1 infection and B*57 in HCV infection. We show, using multiple independent measures, that for both HCV and HTLV-1, possession of the *KIR2DL2* gene enhanced HLA class I-restricted immunity.

## Results

Two independent cohorts were studied: HCV-infected individuals (N = 782) and HTLV-1-infected individuals (N = 402). Ten KIR genes were typed; of these, 4 were present at an informative frequency in the HTLV-1 cohort and 9 in the HCV cohort (defined in Methods). The modulation of HLA class I associations by the informative KIRs was analysed; the models included all other known determinants of outcome in the cohorts as covariates.

### HTLV-1 and disease status

**Figure 1:**
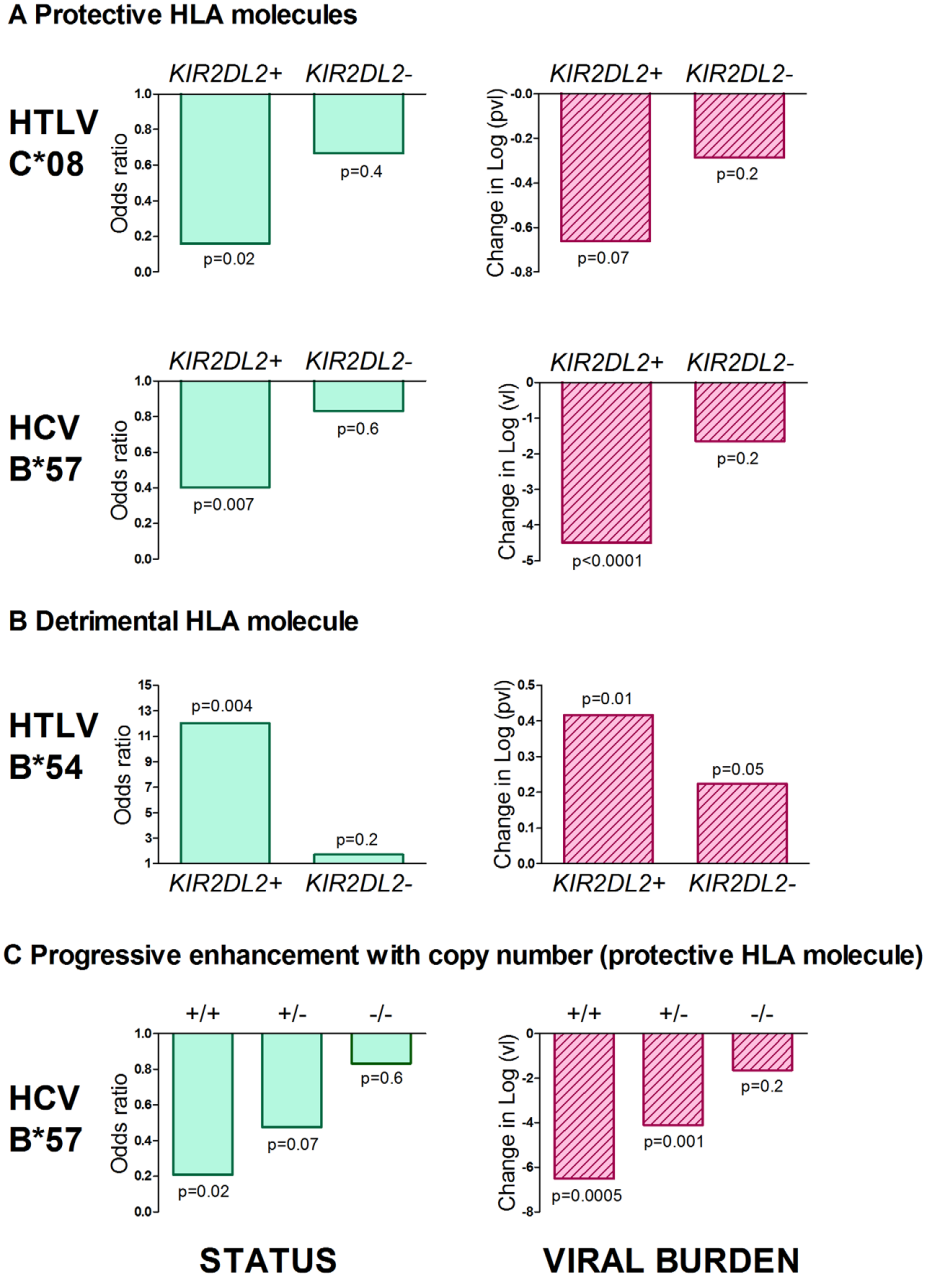
*KIR2DL2* enhances HLA class I mediated antiviral-immunity. The effect of both protective (Figure 1a) and detrimental (Figure 1b) HLA class I alleles on both status (first column) and viral load (second column) is stronger in *KIR2DL2+* individuals than in *KIR2DL2-* individuals. This effect was seen in both HTLV-1 and HCV infection. We also saw a progressive effect of *KIR2DL2* copy number on both status and viral load in HCV (Figure 1c). We could not test for a progressive effect in the HTLV-1 cohort because there are no *KIR2DL2* homozygotes. In all cases, the impact on viral burden was considered within (not between) disease status categories and so the observation of an impact on viral burden is independent of the observation of an impact on status. Effect sizes, p values and cohort sizes are provided in Table 1.

In the cohort from Southern Japan, HLA-C*08 was associated with a significantly reduced odds of developing HAM/TSP (OR =  0.47, p = 0.03, OR<1 indicates a protective effect while OR>1 indicates a detrimental effect)[14]. We investigated the impact of KIRs on this protective effect by stratifying the cohort by KIR genotype. Of the KIR studied, one particular KIR, *KIR2DL2*, had a noticeable interaction with C*08 (Table 1 and Figure 1). We found that the C*08 protective effect was weakened and no longer statistically significant in the subset of individuals who were *KIR2DL2-* (OR = 0.67, p = 0.4) but enhanced in *KIR2DL2+* individuals (OR = 0.16, p = 0.02). There were more *KIR2DL2*- individuals than *KIR2DL2*+ individuals so the absence of significance in the *KIR2DL2-* individuals was not simply due to reduced cohort size. Similarly, *HLA*-B*54, which is associated with a significantly increased risk of HAM/TSP (OR = 3.11, p = 0.0009), had a weakened impact on disease risk in the absence of *KIR2DL2* (OR = 1.70, p = 0.2) but an enhanced impact in the presence of *KIR2DL2* (OR = 12.05, p = 0.004). Again, the absence of a significant effect of B*54 in *KIR2DL2*- individuals was not attributable to a loss of power. In contrast, although HLA-A*02 was associated with a reduced risk of HAM/TSP, there was no significant additional impact of KIR genotype which could not be attributed to power.

**Table 1:** *KIR2DL2* in HTLV-1 infection: *KIR2DL2* enhances the protective effect of C*08 and the detrimental effect of B*54 on HAM/TSP risk and, independently, on proviral load.

HTLV-1 disease status (HAM/TSP v AC)					
	OR in whole cohort *(p value)*	KIR2DL2 Genotype	OR in stratified cohort *(p value)*	HLA Allele Carriers	Cohort size
C*08	0.470 *(p = 0.03)*	+	0.160 *(p = 0.02)*	14	102
		−	0.665 *(p = 0.4)*	44	300
B*54	3.106 *(p = 0.0009)*	+	12.048 *(p = 0.004)*	21	102
		−	1.701 *(p = 0.2)*	64	300

HTLV-1 proviral load						
		Difference in VL in whole cohort *(p value)*	KIR2DL2 Genotype	Difference in VL in stratified cohort *(p value)*	HLA Allele Carriers	Cohort size
C*08	AC	−0.330 *(p = 0.05)*	+	−0.660 *(p = 0.07)*	10	48
			−	−0.286 *(p = 0.2)*	26	132
	HAM/TSP	−0.173 *(p = 0.2)*	+	−0.856 *(p = 0.005)*	4	54
			−	−0.087 *(p = 0.6)*	18	168
B*54	AC	0.057 *(p = 0.8)*	+	−0.218 *(p = 0.7)*	3	48
			−	0.095 *(p = 0.7)*	21	132
	HAM/TSP	0.240 *(p = 0.01)*	+	0.417 *(p = 0.01)*	18	54
			−	0.224 *(p = 0.05)*	43	168

In the disease status models an odds ratio (OR)<1 indicates a protective effect (decreased risk of HAM/TSP), an OR>1 indicates a detrimental effect (increased risk of HAM/TSP). In the proviral load models the dependent variable was log10[proviral load], a difference in VL<0 indicates a protective effect (decreased pvl with the HLA allele), a difference in VL>0 indicates a detrimental effect (increased pvl with the HLA allele). All models also included the two variables which can act as confounding variables in this cohort: age and gender. The impact on proviral load was considered separately in ACs and HAM/TSP and so the observation of an impact on proviral load is independent of the observation of an impact on status. If variables which were not significant predictors (p<0.05) were removed by backwards stepwise exclusion the conclusions were unchanged.

### HTLV-1 and proviral load

As an independent test of the observation that *KIR2DL2* enhanced the effect of both protective and detrimental HLA class I alleles in HTLV-1 infection, we investigated the interaction between HLA class I alleles, *KIR2DL2* and HTLV-1 proviral load (pvl). We investigated pvl in ACs and HAM/TSP patients separately, so any observed impact on pvl is independent of the impact on disease status. C*08 has previously been associated with a low pvl in ACs (difference in log_10_ pvl between C*08+ and C*08- Δ = −0.33, p = 0.05); again, this effect was weakened in *KIR2DL2*- individuals (Δ = −0.29 p = 0.2) but enhanced in *KIR2DL2*+ individuals (Δ = −0.66 p = 0.07); Table 1. Similarly, HLA-B*54, which is associated with a high pvl in HAM/TSP patients (Δ = +0.24 p = 0.01) showed a weakened effect in the absence of *KIR2DL2* (Δ = +0.22, p = 0.05) but an enhanced effect in the presence of *KIR2DL2* (Δ = +0.42, p = 0.01).

Two previous observations on HTLV-1 immunogenetics have, until now, remained unexplained. Firstly, although C*08 has been associated with a low pvl in ACs it has no detectable impact on pvl in HAM/TSP patients; similarly, B*54, which was associated with a high pvl in HAM/TSP patients, had no impact on pvl in ACs [13]–[14]. Why some HLA class I alleles apparently “cease working” in some populations was unknown. We hypothesised that the lack of the expected C*08 and B*54 effects in HAM/TSP patients and ACs respectively was due to a low frequency of *KIR2DL2* in these groups and that the decrease or increase in pvl due to C*08 or B*54 respectively would be manifest only in *KIR2DL2+* individuals. Consistent with this hypothesis we found that the frequency of *KIR2DL2* carriage in the groups that did not show the expected effect of HLA genotype on pvl was approximately half that of the groups in which HLA-associated effects were observed (prevalence of *KIR2DL2+* amongst B*54+ individuals: 12.5% in ACs vs 29.5% in HAM/TSPs; prevalence of *KIR2DL2+* amongst C*08+ individuals: 18.2% in HAM/TSPs vs 27.8% in ACs; Table 1). The small numbers of individuals in the stratified cohorts (HAM/TSP *KIR2DL2+*: C*08+ N = 4, C*08- N = 50. AC *KIR2DL2+*: B*54+ N = 3, B*54- N = 45) precluded a reliable test for an impact of HLA on pvl in *KIR2DL2+* individuals. However, in the larger of these groups there was a significant impact; i.e C*08 was associated with a significant reduction in pvl in *KIR2DL2*+ individuals (Δ = -0.86, p = 0.005). This provides, for the first time, a plausible explanation for the reported observation [14] that the B*54 effect on pvl was not manifest in ACs and the C*08 effect on pvl was not manifest in HAM/TSP patients.

### HTLV-1 and HLA class I specificity

**Figure 2:**
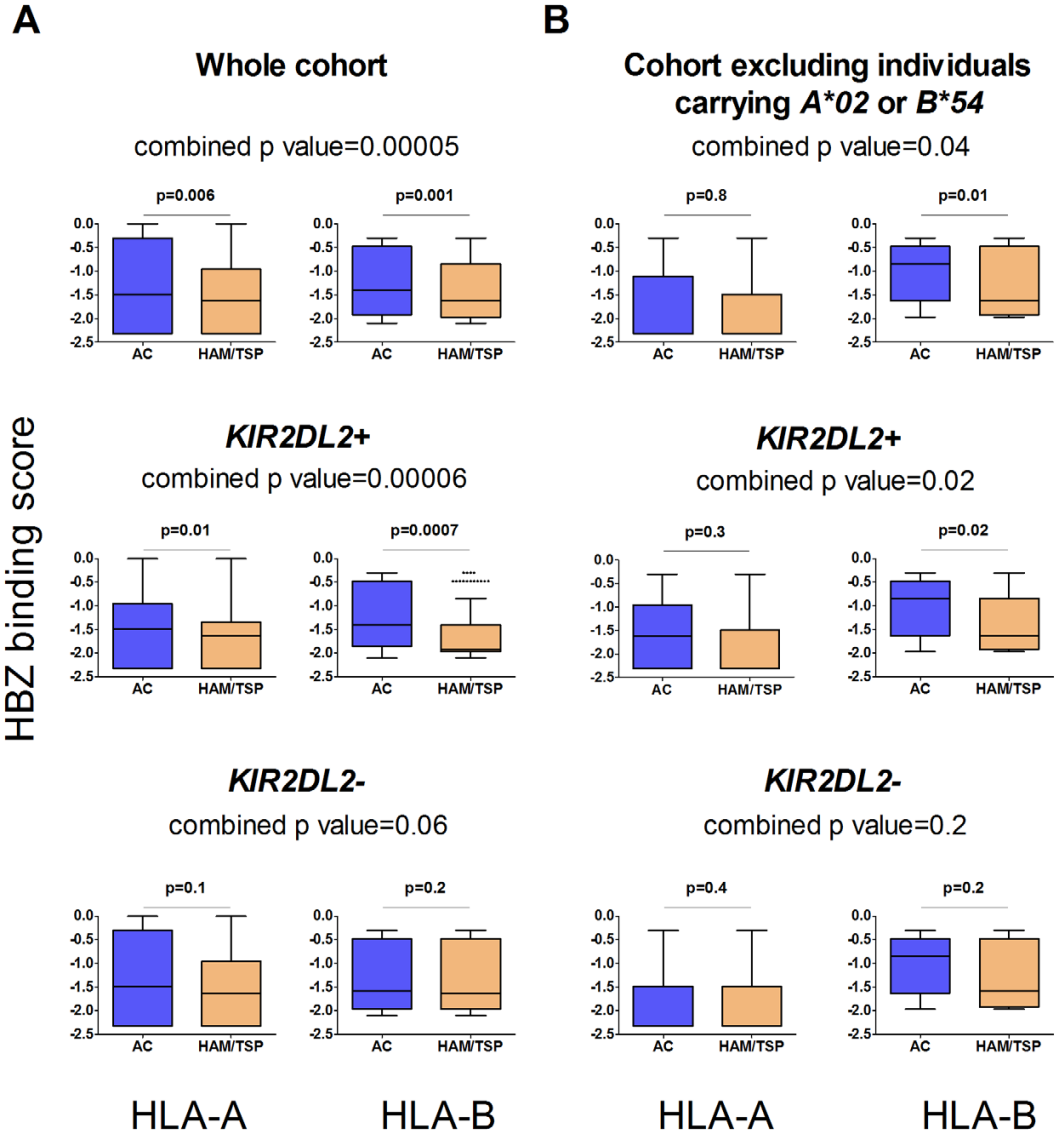
The protective effect of binding HBZ is enhanced by *KIR2DL2*. In the whole cohort (**Figure 2a**) individuals with HLA-A or B molecules which were predicted to bind peptides from HBZ strongly were significantly more likely to be asymptomatic (top row); this effect was stronger in the individuals with *KIR2DL2* (middle row) than without (bottom row). When individuals with A*02 or B*54 were removed (**Figure 2b**) the pattern remained the same. This indicates that the protective effect of binding HBZ peptides by multiple, different HLA class I molecules, both A and B, is enhanced by KIR2DL2.We did not predict HLA-C binding as the relevant algorithms are not available in Metaserver (due to scarcity of HLA C binding data necessary for training the neural networks).

We recently reported that in HTLV-1 infection, HLA class I molecules that bind peptides from the virus protein HBZ are associated with a reduced risk of HAM/TSP and, independently, a reduced pvl [20]. In the same study we showed, using IFNg ELISpot, chromium release and CD107 staining, that HBZ-specific CD8+ T cells were present and functional in fresh PBMC from infected individuals. We therefore investigated the interaction between *KIR2DL2* and the protective effect of binding HBZ. We used epitope prediction software [21] to predict the strength of binding of HBZ peptides to an individual's HLA-A and B molecules. We found that ACs had HLA-A and -B molecules that are predicted to bind HBZ significantly more strongly than those in HAM/TSP patients (median difference 12%, p = 0.00005) [20] and that this effect was stronger in *KIR2DL2+* individuals (median difference 25%, p = 0.00006) than in *KIR2DL2-* individuals (median difference 7%, p = 0.06); Figure 2a. We reasoned that this difference in HBZ binding between ACs and HAM/TSP patients was due to HLA-A*02 and B*54, which differ in their HBZ peptide-binding affinities [20] and are associated with different outcomes in HTLV-1 infection. We therefore removed all individuals with A*02 or B*54 from the cohort and repeated the analysis. Surprisingly, we still found the same pattern: possession of HLA molecules that bind HBZ strongly was significantly associated with remaining asymptomatic (median difference 10%, p = 0.04) and this effect was strengthened in *KIR2DL2+* individuals (median difference 23%, p = 0.02) but not in *KIR2DL2-* individuals (median difference 3%, p = 0.2); Figure 2b. This demonstrates that the protective effect of binding HBZ peptides by multiple HLA class I molecules, both A and B, is enhanced by *KIR2DL2*.

### HCV and viral clearance

As previously reported, HLA-B*57 was associated with significantly decreased odds of chronic infection (OR = 0.571, p = 0.02). This protective effect was enhanced in the presence of *KIR2DL2* (OR = 0.40, p = 0.007) but weakened in the absence of *KIR2DL2* (OR = 0.83, p = 0.6) (Table 2). Furthermore, the impact of B*57 was strongest in *KIR2DL2* homozygote individuals (OR = 0.21, p = 0.02), weaker in *KIR2DL2* heterozygote individuals (OR = 0.48, p = 0.07) and absent in *KIR2DL2*-negative individuals (OR = 0.83, p = 0.6). *KIR2DL2* enhanced the association between B*57 and spontaneous clearance independently and with similar strength in both African Americans and Caucasians (Table S1 in Text S1).

**Table 2:** *KIR2DL2* in HCV infection: *KIR2DL2* enhances the protective effect of HLA-B*57 on HCV status (spontaneous clearance v chronic infection) and, independently, on HCV viral load.

HCV status (spontaneous clearance v chronic infection)				
OR in whole cohort *(p value)*	KIR2DL2 Genotype	OR in stratified cohort *(p value)*	HLA B*57 Allele Carriers	Cohort size
0.571 *(p = 0.02)*	+	0.403 *(p = 0.007)*	49	408
	−	0.832 *(p = 0.6)*	35	374
	+/+	0.209 *(p = 0.02)*	16	84
	−/+	0.475 *(p = 0.07)*	33	324
	−/−	0.832 *(p = 0.6)*	35	374

HCV Viral load								
Difference in VL in whole cohort *(p value)*		KIR2DL2 Genotype	Difference in VL in stratified cohort *(p value)*		HLA B*57 Allele Carriers		Cohort size	
MHCS	ALIVE		MHCS	ALIVE	MHCS	ALIVE	MHCS	ALIVE
−3.106 *(p = 0.0003)*	−0.180 *(p = 0.3)*	+	−4.485 *(p<0.0001)*	−0.459 *(p = 0.05)*	5	13	46	76
		−	−1.640 *(p = 0.2)*	+0.324 (*p = 0.4)*	5	5	25	69

In the HCV status models an odds ratio (OR)<1 indicates a protective effect (decreased odds of chronic infection), an OR>1 indicates a detrimental effect (increased odds of chronic infection). In the viral load models the dependent variable was log10[viral load]. Cohorts were analysed separately as viral load was measured using different assays. Two cohorts (MHCS and ALIVE) had sufficient numbers of individuals with measurements of all variables for this analysis. A difference in VL<0 indicates a protective effect (decreased vl with the HLA allele), a difference in VL>0 indicates a detrimental effect (increased vl with the HLA allele). All models included all variables which can act as confounders (see Methods). The impact on viral burden was considered within chronic carriers and so the observation of an impact on viral burden is independent of the observation of an impact on status. The combined p-values of the difference in VL for the two cohorts are *p_comb_ = 0.00003 in* KIR2DL2+ and *p_comb_ = 0.66* in KIR2DL2-.

### HCV and viral load

**Figure 3:**
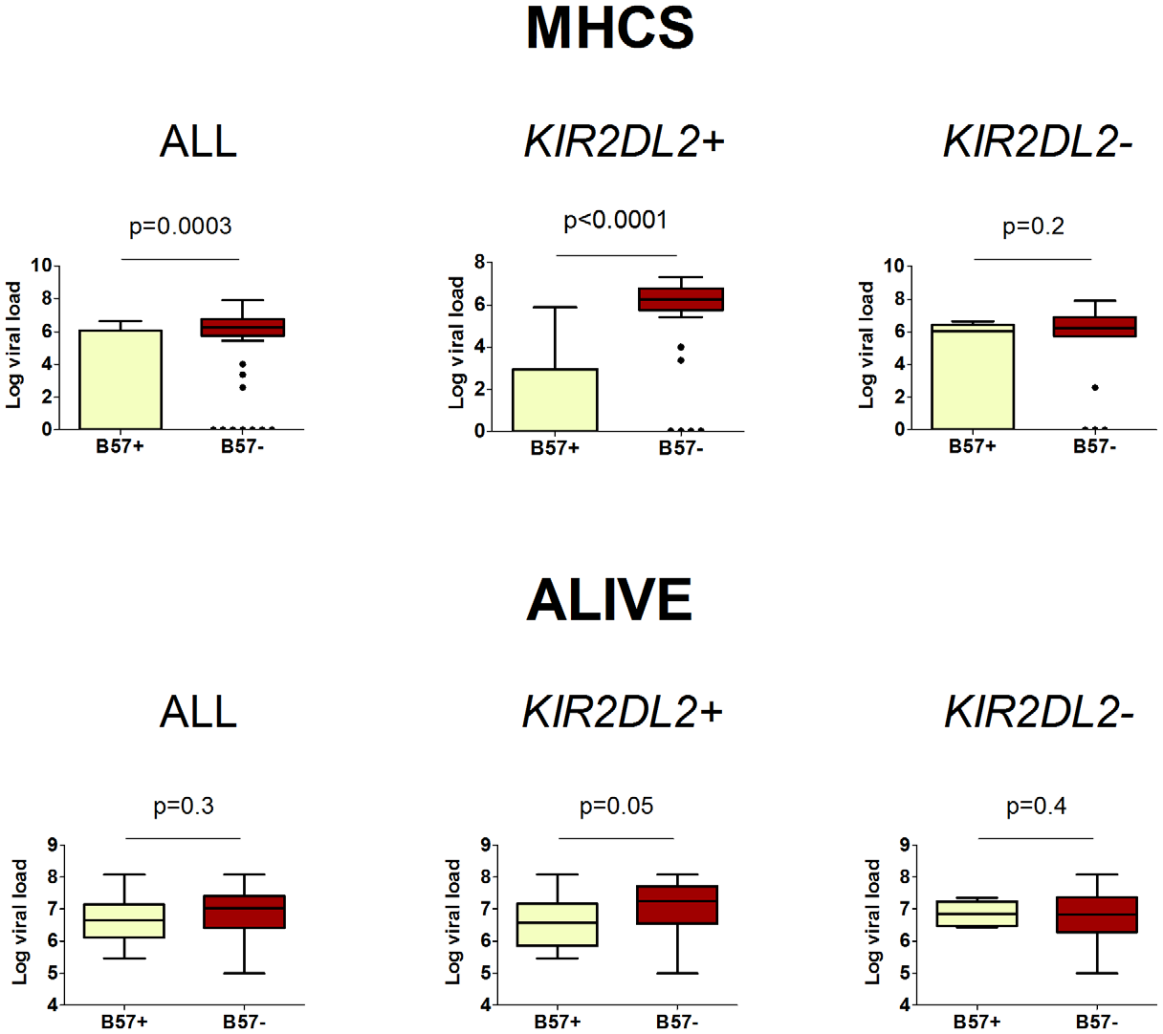
The impact of HLA-B*57 and *KIR2DL2* on HCV viral load. In HCV infection, B*57 is associated with a reduced HCV viral load. This protective effect is enhanced in *KIR2DL2+* individuals and reduced or absent in *KIR2DL2-* individuals. The cohorts were analysed separately as viral load was measured using different assays. Two cohorts, MHCS and ALIVE, had enough individuals with measurements for all factors to perform this analysis. HIV-1 status was a significant determinant of viral load in the ALIVE cohort, including HIV status in the model as a covariate did not change any of our conclusions (ALL *p* = 0.3; KIR2DL2+ *p* = 0.05; KIR2DL2- *p* = 0.4). Summary data from these plots is provided in Table 2. The p-values are derived based on the regression model.

Next we investigated the impact of B*57 and *KIR2DL2* on HCV viral load. We only considered patients with chronic infection, so any observed impact on viral load is independent of the impact on viral clearance. This analysis was possible in two cohorts: MHCS and ALIVE. We found that B*57 was associated with reduced chronic HCV viral load, particularly in the MHCS cohort (MHCS: difference in log10 VL Δ = −3.1 p = 0.0003; ALIVE: Δ = −0.18 p = 0.3. Combined p = 0.0006). Consistent with our observations in HTLV-1 infection, this reduction was enhanced in the presence of *KIR2DL2* (MHCS: Δ = −4.5 p<0.0001, ALIVE: Δ = −0.46 p = 0.05. Combined p = 0.00003) but weakened in the absence of *KIR2DL2* (MHCS Δ = −1.64 p = 0.2, ALIVE Δ = +0.32 p = 0.3. Combined p = 0.7); Table 2 and Figure 3. In MHCS (but not ALIVE) we also observed a progressive effect with *KIR2DL2* copy number (2 copies: Δ = −6.5, p = 0.0005. 1 copy: Δ = −4.1 p = 0.001. 0 copies Δ = −1.6 p = 0.2); the number of homozygous individuals are too small to draw firm conclusions but this progressive effect is consistent with our other observations.

These data show that *KIR2DL2* enhances both protective and detrimental HLA class I associations. Therefore, *KIR2DL2* would not be predicted to have a significant net impact across all HLA class I molecules. That is, possession of *KIR2DL2* (alone or with its C1 ligand) without a particular protective or detrimental HLA allele, would not be expected to be significantly protective or detrimental. This prediction was verified (Table S2 in Text S1).

There is strong linkage disequilibrium among the KIR genes and among the HLA class I genes. Analysis of the linked genes indicates that the primary genes driving the observed associations are most likely to be *KIR2DL2* in combination with HLA-B*54, C*08 and B*57 rather than individual linked KIR, multiple stimulatory linked KIRs or linked HLA class I genes (sections 3 and 4 in Text S1). We cannot rule out an effect of linkage between *KIR2DL2* and neighbouring loci outside the KIR genes. However, there is little evidence of significant linkage between KIRs and even the next closest gene cluster, the LILR [22]. Furthermore, we observed the same effect of *KIR2DL2* in three different populations (Japanese, African-American and Caucasian) so a putative linked locus driving the effect would have to be linked to *KIR2DL2* in all three populations.

### Canonical KIR-HLA binding

Although HLA-C*08, as a group C1 molecule, is expected to bind KIR2DL2, the most frequent subtype in our cohort (Cw*0801, 88%) binds KIR2DL2 very weakly (comparable to background [23]), furthermore HLA-B*54 and HLA-B*57 are not expected to bind KIR2DL2 and the most frequent subtypes in our cohorts (B*5401 and B*5701) have been shown not to bind KIR2DL2 [9], [23]. Finally, KIR2DL2 enhanced the protective effect of binding HBZ peptides by multiple HLA-A and –B molecules. With the exception of B*4601 and B*7301 (which were not responsible for the enhancement, data not shown) KIR2DL2 is not thought to bind HLA-A and B molecules and has been shown not to bind 29 HLA-A and 54 HLA-B allotypes [9]. We therefore hypothesised that the effect of KIR2DL2 on HLA class I-mediated immunity we have observed is not attributable to KIR2DL2 directly binding the HLA molecule whose effect is enhanced. To test this hypothesis we first investigated whether the other group *C1* alleles had the same effect as C*08 in HTLV-1 infection. Grouping all the *C1* alleles we found no significant association between *C1* and decreased risk of HAM/TSP either in the whole cohort or in *KIR2DL2*+ individuals. Similarly, there was no relation between pvl and *C1* in either ACs or HAM/TSP patients. Analysis of the individual *C1* alleles confirmed the hypothesis that the C*08 effect we observed was not exhibited by other group *C1* alleles (Tables S4 and S5).

HLA-B*54, a group *Bw6* HLA allele, is not known to bind any KIR molecule. We therefore tested whether the observed B*54 effect was attributable to C*01, which is in linkage disequilibrium with B*54 and which encodes molecules that bind KIR2DL2. This analysis suggested that B*54, not C*01, was the gene driving the observed detrimental effect on HTLV-1 outcome (section 5.2 in Text S1). This result, and the observation that no other *C1* allele shows “B*54-like” behaviour, indicate that, as postulated, the interaction between B*54 and *KIR2DL2* cannot be explained by direct KIR-HLA binding.

The most frequent B*57 allele in our cohort is B*5701, which does not bind KIR2DL2 [9]. There are therefore two ways in which the observed interaction between KIR2DL2 and HLA-B*57 could be attributed to “classical” KIR-HLA binding: either KIR2DL2 might bind a class I HLA molecule whose encoding gene is linked to HLA-B*57, or the effect might be due to KIR3DL1/S1, which does bind B*57. Analysis of both these possibilities indicated that they did not explain the KIR2DL2-B*57 effect (section 5.3 and 5.4 in Text S1).

Therefore, as hypothesised, the enhancement of C*08, B*54 and B*57–restricted immunity by KIR2DL2 is not explained by direct binding between the respective HLA molecules and KIR2DL2. Instead, we suggest that KIR2DL2 binds its HLA-C ligands and indirectly modulates C*08, B*54 and B*57–restricted T cells. Consistent with this, we found some evidence that KIR2DL2 enhanced HLA Class I effects more strongly when it's stronger C1 ligands are present (section 6 in Text S1).

### Other inhibitory KIR

It seems unlikely that *KIR2DL2* behaves fundamentally differently to other inhibitory KIRs. The effect of *KIR2DL2* may be most apparent because KIR2DL2 is present at informative frequencies and its C1 and C2 ligands are ubiquitous. We addressed the role of other inhibitory KIRs in 3 ways. We studied the effect of individual inhibitory KIRs (section 4.1 in Text S1), we investigated whether the number of inhibitory KIR:ligands had a cumulative effect (section 7 in Text S1) and we examined the role of the group A KIR haplotypes which are dominated by inhibitory KIRs but do not contain *KIR2DL2* (section 8 in Text S1). We found little evidence that the other inhibitory KIRs enhanced HLA class I-mediated immunity but this may be due to small cohort sizes and masking by the dominant *KIR2DL2* effect.

### Activating KIR

We found no evidence that activating KIR were enhancing HLA class I-restricted immunity. Haplotype B, the more activatory KIR haplotype, enhanced HLA class I associations but this was only true if the haplotype contained KIR2DL2 (section 8 in Text S1). We found no evidence that the cumulative presence of activating KIR enhanced HLA class I restricted immunity (section 4.3 in Text S1). And, as far as it was possible to separate KIR2DL2 and KIR2DS2, which are in tight linkage disequilibrium, the enhancement of HLA class I restricted immunity appeared to be attributable to KIR2DL2 rather than KIR2DS2 (section 4.2 in Text S1).

## Discussion

We show that *KIR2DL2* enhanced several independent HLA class I-mediated effects in two unrelated viral infections. In HTLV-1 infection, *KIR2DL2* enhanced the protective and detrimental effects of HLA-C*08 and B*54 respectively on disease status. *KIR2DL2* also enhanced the association between C*08 and low proviral load in ACs and between B*54 and high proviral load in HAM/TSP patients. Additionally, *KIR2DL2* enhanced the protective effect of HBZ binding by multiple HLA molecules. Strikingly, on stratifying by *KIR2DL2*, we observed, for the first time, a protective effect of C*08 on pvl in HAM/TSP patients and explained the lack of impact of B*54 on pvl in ACs. In HCV infection, *KIR2DL2* enhanced the protective effect of B*57 on spontaneous clearance and the association between B*57 and low viral load in chronic carriers; for both clearance and viral load a progressive effect with *KIR2DL2* copy number was observed. This progressive effect is consistent with reports of an association between KIR gene copy number and the frequency of cell-surface expression of the respective KIR molecule [24]–[25].

There are two mechanisms by which KIR2DL2 could act: it could enhance either NK-mediated or T cell-mediated immunity. That is, NK cell killing of virus-infected cells could be altered by KIR2DL2 expression or, alternatively, the virus-specific CD8+ T cell response could be modified by KIR2DL2 expression (on NK cells or T cells). Two observations indicate that it is the T cell response that is more likely to be enhanced. First, strong binding of HBZ viral peptides via multiple different HLA-A and B molecules was associated with asymptomatic status [20] and this protective effect was enhanced by *KIR2DL2*. KIR2DL2 is not known to bind HLA-A or–B molecules (with the exception of B*4601 and B*7301) [9], [23], [26] so it is unlikely that the enhancement of the protective effect of binding HBZ by KIR2DL2 is due to direct binding between KIR2DL2 and HBZ peptide in the context of HLA-A and –B molecules. Furthermore, although NK cells exhibit peptide dependence [27], it is hard to reconcile protein-specificity via multiple HLA molecules with an NK cell-mediated mechanism. Second, the *KIR2DL2* enhancement could not be explained by binding between KIR2DL2 and any of the 3 HLA class I molecules investigated. One further observation also suggests a T cell-mediated mechanism. Two protective genotypes in HCV infection that are postulated to operate via innate immune mechanisms [28] (namely a SNP upstream of *IL28B* and *KIR2DL3-HLA-C1)* had no impact on viral load in chronic infection [10], [29]. The authors hypothesised that this was because innate barriers offer little protection once overcome. In contrast, the KIR2DL2/B*57 effect that we report here had a significant impact on viral load: again, this is perhaps more consistent with adaptive immunity.

**Figure 4:**
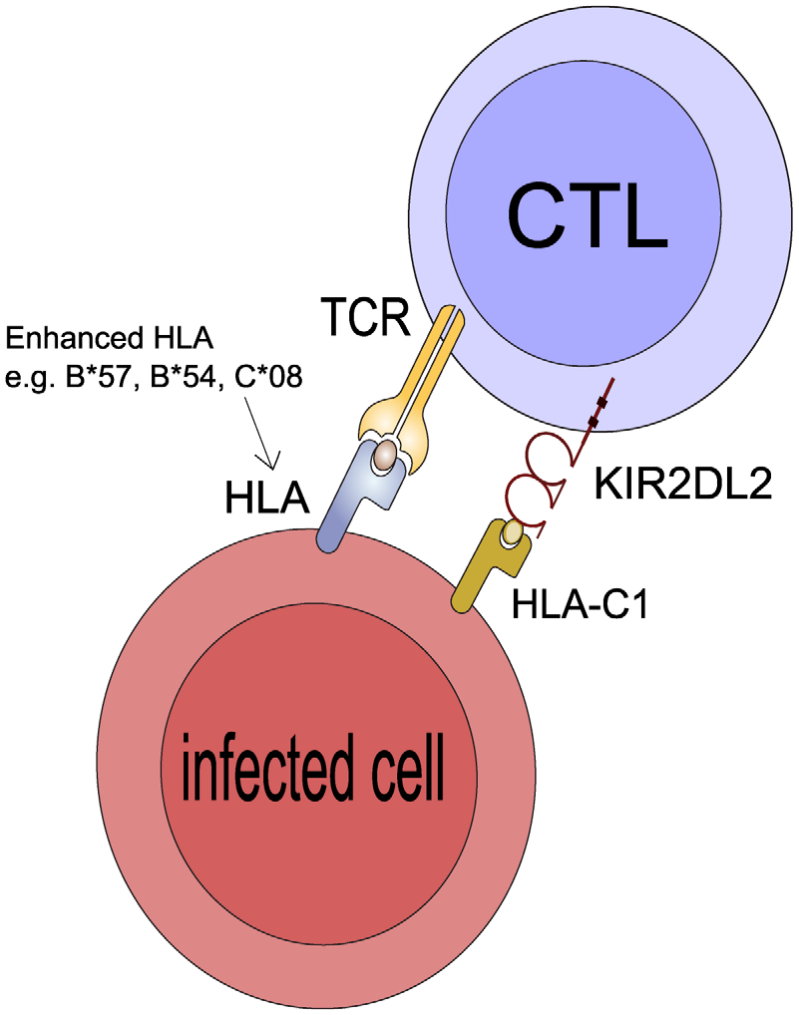
Postulated mechanism: KIR2DL2 reduces clonal exhaustion of CD8+ T cells and is necessary for an effective immune response in the face of chronic antigen stimulation. It has been demonstrated that CD8+ T cells that express inhibitory KIR have elevated levels of Bcl-2, are less susceptible to activation induced cell death (AICD) and have a survival advantage leading to their accumulation in vitro and in vivo [30], [31]. We suggest KIR2DL2 binds its HLA-C ligands so that when the CD8+ T cell is activated by engagement of its TCR by the cognate HLA:peptide complex the CD8+ T cell will less likely to undergo AICD. If the CD8+ T cell is restricted by a protective HLA class I molecule (e.g. B*57 in HCV or C*08 in HTLV-1) it will survive for longer than in a person who is KIR2DL2- and its protective effects will be enhanced. Similarly, if the CD8+ T cell is restricted by a detrimental HLA molecule (e.g. B54 in HTLV-1) it will also survive for longer and its detrimental effects will be enhanced. Of note, the HLA molecule whose protective/detrimental effects are enhanced is the HLA molecule that binds the TCR not the HLA that binds KIR2DL2. This postulated mechanism explains 3 striking features of our observations: i) that KIR2DL2, a receptor typically associated with innate immunity, enhances adaptive immunity ii) that the effect cannot be explained by KIR2DL2 binding the enhanced HLA molecules directly iii) that both protective and detrimental HLA effects are enhanced.

Our results indicate that *KIR2DL2* enhances HLA class 1-restricted CD8+ T cell-mediated adaptive immunity. KIRs on both NK cells and CD8+ T cells have been reported to shape adaptive immunity [5]-[7]. Of particular interest are reports [30]–[33] that inhibitory KIRs on CD8+ T cells promote the survival of a subset of memory phenotype CD8+ αβ T cells with enhanced cytolytic potential (Tm1 [34]) by reducing activation-induced cell death. Ugolini et al suggested that this phenomenon helps maintain specific CD8+ T lymphocytes during chronic viral infection [30]. Tm1 cells have been described in both HTLV-1 and HCV infections, where they constitute a minority of virus-specific CD8+ T cells but the majority of perforin-bright cells [35]–[36]. Consistent with our findings, these studies showed that the HLA molecule that restricts the T cell whose survival is promoted was independent of the HLA-C molecules that ligated the KIR [30], [34]. We postulate that, in the face of chronic antigen stimulation, protective T cells survive longer if they carry *KIR2DL2* and therefore exert stronger protection. Likewise, T cells restricted by HLA alleles associated with increased disease susceptibility also survive for longer in the presence of *KIR2DL2* and so are more detrimental (Figure 4). Hence, *KIR2DL2* enhances both protective and detrimental HLA class I associations.

Alternatively, it is known that NK cells kill activated T cells and that this killing is reduced by inhibitory KIR [37]–[38]. So again, T cells restricted by protective and detrimental HLA class I molecules may survive longer in the presence of inhibitory KIR and thus the protective and detrimental associations would be enhanced.

Ugolini et al proposed that inhibitory KIRs promote T cell survival by increasing the activation threshold of T cells. This may explain why the HLA-A*02 protective effect in HTLV-1 is not significantly enhanced by *KIR2DL2*. A*02 molecules bind peptides significantly more strongly than other alleles (section 9 in Text S1) and the immunodominant HTLV-1 peptide Tax 11-19 is bound exceptionally strongly. Therefore, even if the T cell activation threshold were increased, the strength of signalling may remain above the threshold and consequently the A*02 protective effect cannot be enhanced.

Why does *KIR2DL2* enhance T cell responses whereas the other inhibitory KIRs apparently do not? The effect of *KIR2DL2* may be most apparent because *KIR2DL2* is present at informative frequencies and its C1 and C2 ligands are ubiquitous; i.e. unlike the other KIR every individual carries a KIR2DL2 ligand.

It will be important to determine whether inhibitory KIRs play a similar role in enhancing CD8+ and possibly CD4+ T cell-mediated immunity to other pathogens and in autoimmune disease. KIR-expressing virus-specific CD8+ T cells have been reported in other chronic infections including HIV-1, CMV and EBV [39]-[41]. Furthermore, in HIV-1 infection, high expression alleles of an inhibitory KIR, *KIR3DL1*, in the context of *HLA-Bw4I* have been associated with slow progression to AIDS [11]. In order to explain protection by an inhibitory KIR the authors proposed a model based on NK cell development. Our results suggest an alternative explanation, i.e. that *KIR3DL1* enhances protective HLA-B-restricted responses to HIV-1.

In contrast to previous studies of KIR genotype, which investigated the antiviral action of NK cells, we investigated the impact of KIRs on HLA class I-mediated antiviral immunity. We find a clear and consistent effect of *KIR2DL2*. The effect sizes are striking: *KIR2DL2* homozygotes with B*57 are almost 5 times more likely to clear HCV infection spontaneously; if they fail to clear the virus they have a viral load that is reduced by 6.5 logs. Until now, the advantages offered by inhibitory KIRs in virus infections have been unclear. Our data support an alternative role in which inhibitory KIRs enhance both beneficial and detrimental T cell-mediated immunity in persistent viral infection.

## Methods

### Ethics statement

The HTLV-1 cohort has been approved by the following committees: 1) St. Mary's Local Research Ethics Committee, 1995: title “The immunology and virology of the treatment of HTLV-1-associated inflammatory disease”. Approval reference number: EC3108. 2) Kagoshima University Hospital Clinical Research Ethics Committee: 27th May 1999. Title: “Investigation of HAM pathomechanism: relationship between host genetic background and clinical status of HTLV-1 infection”. Approval reference number: 22. All samples were taken under written informed consent.

### Cohorts

The HCV cohort consisted of four sub-cohorts: AIDS Link to Intravenous Experience (ALIVE, N = 262) [42], Multicenter Hemophilia Cohort Study (MHCS, N = 320) [43], Hemophilia Growth and Development Study (HGDS, N = 110) [44] and a UK cohort (N = 341) [10]. 251 individuals were excluded due to incomplete information. The cohort had 257 resolved and 525 chronic patients. HLA class I associations in three of these 4 cohorts (ALIVE, MHCS and HGDS) have previously been reported [18].

The HTLV-1 cohort (N = 431) [14] consists of individuals recruited in Kagoshima Japan. All individuals were of Japanese ethnic origin and resided in Kagoshima Prefecture, Japan. The cohort had 229 HAM/TSP patients and 202 asymptomatic carriers.

### HLA genotyping

HLA genotyping of the HCV and HTLV-1 cohorts was performed in previous studies [10], [14], [18].

#### HCV

Genomic DNA was amplified using locus specific primers as described [10], [18]. The resulting PCR products were blotted onto nitrocellulose membranes and hybridized with sequence specific oligonucleotide probes (SSO). US: alleles were assigned according to the reaction patterns of the SSO probes, ambiguities were resolved by sequencing analysis. UK: PCR products were typed by direct sequencing. HLA types that were not resolved by sequencing or which gave unusual results were also tested by SSO typing using commercial kits (Dynal, RELI SSO, Wirral, UK).

#### HTLV-1

PCR–sequence-specific primer reactions were performed as described [45].

### KIR genotyping

KIR genotyping of the HCV cohort (but not the HTLV-1 cohort) was performed previously [10].

#### HCV

The presence or absence of ten KIR genes (*KIR2DL1, KIR2DL2, KIR2DL3, KIR3DL1, KIR2DS1, KIR2DS2, KIR2DS3, KIR2DS4, KIR2DS5* and *KIR3DS1*) was determined by PCR using sequence specific primers as described in [46].

#### HTLV-1

The presence or absence of ten KIR genes (*KIR2DL1, KIR2DL2, KIR2DL3, KIR2DL4, KIR3DL1, KIR2DS2, KIR2DS3, KIR2DS4, KIR2DS5* and *KIR3DS1*) was determined by PCR using sequence specific primers [47]. 30 of the 431 individuals were not KIR genotyped due to insufficient DNA.

This method of KIR typing does not allow the direct determination of *KIR2DL2* copy number. Instead we use the allelic nature of *KIR2DL2* and *KIR2DL3* at the *2DL2/2DL3* locus to infer the number of copies of *KIR2DL2*.

### Viral load

#### HCV

HCV Cohorts were analysed separately as viral load was measured using different assays. Two cohorts (MHCS and ALIVE) had sufficient numbers of individuals with measurements of all variables for this analysis. For MHCS we used the median of multiple measurements of viral load; for ALIVE, a single measurement was available for each subject. We only analysed viral load in patients with chronic infection, so any observed impact on viral load is independent of an observed impact on viral clearance.

HCV RNA was assessed by branched DNA (Quantiplex HCV RNA 2.0 assay; Chiron Corporation) [ALIVE] or the HCV COBAS AMPLICOR system (COBAS AMPLICOR HCV; Roche Diagnostics) [MHCS].

#### HTLV-1

The HTLV-1 provirus load in peripheral blood mononuclear cells (PBMC) was measured as described in [48]. Quantitative PCR was performed using an ABI 7700 sequence detector (Perkin–Elmer Applied Biosystems). All DNA standards and samples were amplified in triplicate. A standard curve was generated by using the β-actin gene from HTLV-1-negative PBMC and the Tax gene from TARL-2, a cell line containing a single copy of HTLV-1 proviral DNA. We analysed viral load separately in ACs and HAM/TSP patients, so any observed impact on viral load is independent of any observed impact on disease status.

### Statistical analysis

#### HLA class I

HLA class I alleles which were significantly associated with outcome in our cohorts and that were independently verified (either in a large independent cohort or on an independent outcome) were studied. For HTLV-1 there are three such associations: HLA-A*02 and C*08 which are associated with reduced proviral load in ACs and reduced risk of HAM/TSP and HLA-B*54 which is associated with increased proviral load in HAM/TSP patients and increased risk of HAM/TSP [13]-[14]. For HCV there are two such associations: HLA-B*57 and C*01 which have both been associated with increased odds of viral clearance in two independent cohorts [17], [49]. The C*01-protective effect in HCV infection has been shown to be NK-mediated [10], [28] and therefore we did not consider it here.

#### KIR

We investigated KIR genes which were present at an informative frequency. We define an informative frequency as sufficient to detect a “moderate” effect size (δ = 0.5, [50]); this is equivalent to at least 32 people carrying the gene and 32 not carrying the gene. Of the KIR genes typed, 4 were present at an informative frequency in the HTLV-I cohort (*KIR2DL2, KIR2DS2, KIR2DS3* and *KIR3DS1)* and 9 in the HCV cohort (*KIR2DL2, KIR2DL3, KIR3DL1, KIR2DS1, KIR2DS2, KIR2DS3, KIR2DS4, KIR2DS5* and *KIR3DS1*).

The effect of individual HLA class I alleles in different KIR genetic background was investigated by stratifying the cohorts for the absence and presence of the informative KIRs and the effect of the already described HLA class I associations were re-evaluated in each stratum. The impact of the HLA alleles on two response variables was studied: 1) status (AC vs. HAM/TSP for HTLV-1 infection and resolved vs. chronic for HCV infection) and 2) viral burden (log10[proviral load] for HTLV-1 infection and log10[viral load] for HCV infection). Multiple logistic regression was used to study the variation in status and multiple linear regression was used for the variation in viral burden. All statistically significant confounding variables were included in the models (HTLV-1: age and gender; HCV: HBV status, mode of infection, SNP rs12979860 and cohort). We focused on the difference between KIR+ and KIR- individuals in the size of the protective/detrimental effects associated with the individual HLA class I molecules (i.e. odds ratios and differences in viral load) rather than p values as the latter comparison is confounded by differences in strata sizes.

We quantified the false discovery rate for our analysis using Monte Carlo methods. For each of the HLA associations studied, we performed 10,000 random stratifications of the relevant (HCV or HTLV-1) cohort with the size of the two strata being the number of KIR2DL2+ and KIR2DL2- individuals in that cohort and asked how many times we would see odds ratios equal to or more extreme than we observed in the actual cohorts. The probability of making our observations by chance is less than p = 2×10^−11^.

Linkage disequilibrium (LD) between HLA class I alleles was calculated using the tool available at www.hiv.lanl.gov. The LD between KIRs was calculated based on the Chi-squared test on a 2x2 contingency table. All the reported p values are two-tailed. Where applicable, independent p values were combined using Fisher's combined test. Statistical analysis was performed using R v2.9.2.

### Epitope prediction

The binding strength of HLA class I molecules to viral proteins was assessed using epitope prediction software. Prediction of T cell class I epitopes is now highly accurate and algorithms can achieve accuracy of up to 94% [51]. In this study we use the epitope prediction software Metaserver [21] (http://web.bioinformatics.ic.ac.uk/metaserver).

### Rank measure for predicted epitopes

We used the rank measure technique [52] in which the strength of an allele's preference for a particular protein is quantified by ranking the strength of binding of the top binding peptide from the protein of interest amongst the strength of binding of peptides from the entire proteome to that allele. Specifically, we split the HTLV-1 proteome into overlapping nonamers offset by a single amino acid and predicted a binding affinity score for each nonomer. For each allele we rank all nonamers from the proteome from the weakest to strongest predicted binding scores. This produces a list of rank values for each protein to that particular allele that quantifies the binding relationship between that allele and the protein. We then invert the rank so the bigger 1/rank, the stronger the preference of the allele for the protein; the logarithm of this measure is plotted on the y axis of Figure 2 as “HBZ binding score”. Each individual therefore contributes up to 4 values (alleles for which no predictive algorithms were available were excluded from the analysis). Binding scores were compared between ACs and HAM/TSP patients using the Wilcoxon rank sum test and reported both as separate p values for HLA-A and B molecules and combined (since we found no evidence to reject the null hypothesis that the HBZ binding score of an individual's A and B molecules was independent, spearman correlation = 0.05 p = 0.5). The median difference in binding score is the median of the difference of average HBZ binding between ACs and HAM/TSP patients expressed as a percent of the AC binding score for HLA-A and –B molecules.

## Supporting Information

Text S1 **Additional analysis and results.** Supporting information on impact of race, KIR and HLA linkage disequilibrium, canonical KIR-HLA binding, KIR haplotypes, other inhibitory and activatory KIRs and other additional results.
(DOC)
