# Supplementary material for: KIR2DL2 Enhances Protective and Detrimental HLA Class I-Mediated Immunity in Chronic Viral Infection
Source: PLoS Pathog. 2011 Oct 13;7(10):e1002270. doi: 10.1371/journal.ppat.1002270 (PMC3192839; doi:10.1371/journal.ppat.1002270)
Supplement: Text S1 — Additional analysis and results. Supporting information on impact of race, KIR and HLA linkage disequilibrium, canonical KIR-HLA binding, KIR haplotypes, other inhibitory and activatory KIRs and other additional results. (DOC) [file ppat.1002270.s001.doc]

# Supporting Information

Table of Contents

[1. *KIR2DL2* enhancement of the *B*57* protective effect in HCV infection is seen in both Caucasians and African Americans 2](#__RefHeading___Toc297735469)

[2. *KIR2DL2* on its own or with *C1* is not associated with status or viral burden 3](#__RefHeading___Toc297735470)

[3. HLA linkage 3](#__RefHeading___Toc297735471)

[*3.1.* HTLV-1: *C*08* rather than linked HLA genes appears to be the primary gene driving protection which is enhanced by *KIR2DL2* 3](#__RefHeading___Toc297735472)

[3.2. HTLV-1: *B*54* rather than linked HLA genes appears to be the primary gene driving susceptibility which is enhanced by *KIR2DL2* 4](#__RefHeading___Toc297735473)

[3.3. HCV: *B*57* rather than linked HLA genes appears to be the primary gene driving protection which is enhanced by *KIR2DL2* 4](#__RefHeading___Toc297735474)

[4. KIR linkage 5](#__RefHeading___Toc297735475)

[4.1. *KIR2DL2* rather than a linked KIR appears to be the primary KIR enhancing both the protective and detrimental associations with HLA class I in HTLV-I and HCV infection. 5](#__RefHeading___Toc297735476)

[4.2. KIR2DL2 or KIR2DS2? 7](#__RefHeading___Toc297735477)

[4.3. The cumulative presence of stimulatory receptors 8](#__RefHeading___Toc297735478)

[5. Canonical KIR-HLA binding 8](#__RefHeading___Toc297735479)

[5.1. HTLV-1: other group *C1* HLA alleles do not exhibit the same behaviour as *C*08* 8](#__RefHeading___Toc297735480)

[5.2. HTLV-1: the *B*54* effect cannot be attributed to linkage with *C*01* 10](#__RefHeading___Toc297735481)

[5.3. HCV: the *B*57-KIR2DL2* effect cannot be attributed to *B*57* linkage with HLA molecules that do bind KIR2DL2 10](#__RefHeading___Toc297735482)

[5.4. HCV: the *B*57-KIR2DL2* effect cannot be attributed to *KIR2DL2* linkage with other KIRs that do bind B*57 10](#__RefHeading___Toc297735483)

[6. Does the effect of KIR2DL2 depend on the presence of its ligands? 12](#__RefHeading___Toc297735484)

[7. The cumulative presence of inhibitory KIRs 12](#__RefHeading___Toc297735485)

[8. The role of KIR haplotypes 13](#__RefHeading___Toc297735486)

[9. A*02 binds peptides strongly 14](#__RefHeading___Toc297735487)

# *KIR2DL2* enhancement of the *B*57* protective effect in HCV infection is seen in both Caucasians and African Americans

There was a trend for *B*57* to be associated with an increased odds of HCV clearance in both African-Americans and in Caucasians. On stratifying the cohorts by *KIR2DL2* genotype this trend became significant for *KIR2DL2+* individuals but was lost for *KIR2DL2-* individuals. The same pattern and a similar strength of effect were seen in both African-Americans and Caucasians.

| Unstratified cohort | | | | | | |
| --- | --- | --- | --- | --- | --- | --- |
| Cohort | OR | LCI | UCI | p-value | Allele carriers | Cohort size |
| All | 0.569 | 0.351 | 0.923 | 0.023 | 84 | 782 |
| African-Americans | 0.482 | 0.222 | 1.046 | 0.065 | 34 | 240 |
| Caucasians | 0.556 | 0.283 | 1.092 | 0.087 | 43 | 483 |

| *KIR2DL2+* | | | | | | |
| --- | --- | --- | --- | --- | --- | --- |
| Cohort | OR | LCI | UCI | p-value | Allele carriers | Cohort size |
| All | 0.403 | 0.208 | 0.781 | 0.007 | 49 | 408 |
| African-Americans | 0.354 | 0.130 | 0.960 | 0.041 | 25 | 125 |
| Caucasians | 0.363 | 0.134 | 0.985 | 0.047 | 21 | 252 |
| *KIR2DL2-* | | | | | | |
| Cohort | OR | LCI | UCI | p-value | Allele carriers | Cohort size |
| All | 0.832 | 0.392 | 1.764 | 0.632 | 35 | 374 |
| African-Americans | 0.649 | 0.150 | 2.801 | 0.563 | 9 | 115 |
| Caucasians | 0.755 | 0.286 | 3.759 | 0.57 | 22 | 231 |
| **Table S1. *KIR2DL2* enhancement of the protective effect of *B*57* is seen in both Caucasians and African-Americans** | | | | | | |

# *KIR2DL2* on its own or with *C1* is not associated with status or viral burden

|  | *KIR2DL2* | *KIR2DL2:C1* |
| --- | --- | --- |
|  | OR  *(p value)* | |
| HTLV-1 status | 0.90  *(p=0.71)* | 0.88  *(p=0.67)* |
| HCV status | 1.16  *(p=0.34)* | 1.11  *(p=0.51)* |
|  | Difference in logVL  *(p value)* | |
| HTLV-1 pvl AC | 0.05  *(p=0.77)* | 0.05  *(p=0.77)* |
| HTLV-1 pvl HAM/TSP | -0.02  *(p=0.85)* | -0.03  *(p=0.78)* |
| HCV vl MHCS | 0.09  *(p=0.89)* | 1.11  *(p=0.07)* |
| HCV vl ALIVE | 0.22  *(p=0.08)* | 0.09  *(p=0.47)* |
| **Table S2: *KIR2DL2* does not have an effect on status or viral burden, with or without its C1 ligand** | | |

The same result for HCV infection has been reported in [1].

This is consistent with a picture in which *KIR2DL2* enhances both protective and detrimental antiviral responses, so across all responses, it does not have a net (protective or detrimental) effect.

# HLA linkage

## HTLV-1: *C*08* rather than linked HLA genes appears to be the primary gene driving protection which is enhanced by *KIR2DL2*

In the HTLV-I cohort, *C*08* was in linkage disequilibrium with *B*40* and *B*48;* we therefore sought to establish which was the primary HLA class I gene driving the protective association which was enhanced by *KIR2DL2*.

*C*08 & B*40.*

In a logistic regression model to predict disease status (HAM/TSP v AC) when both *C*08* and *B*40* were included as factors *C*08* retained a protective trend (OR=0.52 p=0.07) whereas *B*40* lost significance (OR=0.88 p=0.3); stratifying on *KIR2DL2* we again found that in *KIR2DL2*+ individuals, *C*08* retained a protective trend (OR=0.22 p=0.07) and *B*40* lost significance (OR=0.49, p=0.20) and that, as expected, neither was significant in the absence of *KIR2DL2*. Similarly in a linear regression model to predict log10(proviral load) in ACs when both *C*08* and *B*40* were included as factors *C*08* retained significance (difference in logVL=-0.37 p=0.03) whereas *B*40* lost significance (indeed went in the other direction, difference in logVL=+0.14 p=0.3); stratifying on *KIR2DL2* we again found that in *KIR2DL2*+ individuals the *C*08* effect was strengthened (difference in logVL=-0.64 p=0.07) and *B*40* lost significance (difference in logVL=+0.3, p=0.20) and that, as expected, neither was significant in the absence of *KIR2DL2*. We therefore concluded that *C*08* rather than *B*40* was the HLA gene most likely to be associated with protection whose effect was enhanced by *KIR2DL2*.

*C*08 & B*48.*

In a logistic regression model to predict disease status (HAM/TSP v AC) when both *C*08* and *B*48* were included as factors both factors lost significance. However, stratifying on *KIR2DL2* we found that in *KIR2DL2*+ individuals *C*08* retained a protective trend (OR=0.21, p=0.07) and *B*48* lost significance (OR=0.33, p=0.34) and that, as expected, neither was significant in the absence of *KIR2DL2*. More convincingly, in a linear regression model to predict log10(proviral load) in ACs when both *C*08* and *B*40* were included as factors *C*08* retained a trend (difference in logVL=-0.36 p=0.08) whereas *B*48* lost significance (indeed went in the other direction, difference in logVL=+0.08 p=0.78); stratifying on *KIR2DL2* we again found that in *KIR2DL2*+ individuals the *C*08* effect was strengthened (difference in logVL=-1.1 p=0.03) and *B*40* lost significance (again went in the opposite direction, difference in logVL=+0.7, p=0.24) and that, as expected, neither was significant in the absence of *KIR2DL2*. Together, these data suggest that *C*08* rather than *B*48* was the gene most likely to be associated with protection whose effect was enhanced by *KIR2DL2*.

## HTLV-1: *B*54* rather than linked HLA genes appears to be the primary gene driving susceptibility which is enhanced by *KIR2DL2*

In the HTLV-I cohort, *B*54* was in linkage disequilibrium with *C*01.* We therefore investigated i) whether *B*54* or *C*01* was the primary gene associated with increased susceptibility to HAM/TSP and ii) whether *B*54* or *C*01*-associated susceptibility was enhanced by *KIR2DL2*. In a logistic regression model when both *B*54* and *C*01* were included as factors *B*54* retained significance (OR=3.84 p=0.0009) whereas *C*01* lost significance (indeed went in the opposite direction OR=0.73 p=0.3); stratifying on *KIR2DL2* we again found that in *KIR2DL2*+ individuals *B*54* retained significance and *C*01* lost significance and that, as expected, neither was significant in the absence of *KIR2DL2*. If all *B*54*+ individuals were removed from the cohort then *C*01* was no longer detrimental (OR=0.88, p=0.4, C*01 carriers=96). Unfortunately, due to the large number of *C*01+* individuals in the cohort (N=184) it was not possible to reverse this analysis and investigate the impact of *B*54* in the absence of *C*01.*

## HCV: *B*57* rather than linked HLA genes appears to be the primary gene driving protection which is enhanced by *KIR2DL2*

In the HCV cohort, *B*57* is in linkage disequilibrium with *A*01*, *C*06* and *C*18*. Hence, we investigated whether the observed protective effect of *B*57* can be attributed to the other linked alleles. We found that *A*01*, *C*0*6 and *C*18* do not have a significant impact on disease status neither overall nor in the context of *KIR2DL2*. We therefore conclude that *B*57* is the HLA allele associated with HCV clearance.

# KIR linkage

## *KIR2DL2* rather than a linked KIR appears to be the primary KIR enhancing both the protective and detrimental associations with HLA class I in HTLV-I and HCV infection.

The KIR genes are in tight linkage disequilibrium (Figure S1), making it hard to definitively ascertain which KIR enhances the HLA-associated effects (i.e. the association between *C*08* and asymptomatic status in HTLV-1 infection, between *B*54* and HAM/TSP in HTLV-1 infection and between *B*57* and spontaneous viral clearance in HCV infection). To try to determine which was the primary KIR driving the enhancement of the HLA-associated effects we constructed a logistic regression model to predict status (HAM/TSP v AC for HTLV-1 infection, spontaneous clearance v persistence for HCV infection) in which the HLA molecule with the presence or absence of each KIR, depending on the stratum in which the HLA had the more significant effect, was included along with the known confounding factors. Then, we remove the HLA:KIR factors by stepwise backwards exclusion (i.e. by the highest p-value one at a time, refit the model and repeat). In all 3 cases (*C*08*, *B*54* and *B*57*) the only HLA:KIR compound that remains in the model is the HLA:KIR2DL2+. KIR2DS2, the activating form of KIR2DL2 was also examined more closely (see section 4.2).

Additionally, HLA-KIR factors which may be particularly relevant because they are known receptor-ligand pairs (e.g. HLA B*57 with KIR3DL1 or KIR3DS1) were examined in more detail: see section 5, Canonical KIR:HLA binding.

| <0.001 |  |  |  |  |  | KIR2DL2 |
| --- | --- | --- | --- | --- | --- | --- |
| 0.04 | <0.001 |  |  |  |  | KIR3DL1 |
| <0.001 | 0.76 | <0.001 |  |  |  | KIR2DS2 |
| <0.001 | <0.001 | <0.001 | <0.001 |  |  | KIR2DS3 |
| 0.1 | 0.02 | 0.69 | 0.8 | <0.001 |  | KIR2DS4 |
| <0.001 | <0.001 | <0.001 | <0.001 | 0.27 | <0.001 | KIR3DS1 |
| KIR2DL2 | KIR3DL1 | KIR2DS2 | KIR2DS3 | KIR2DS4 | KIR3DS1 |  |

| <0.001 |  | | | | | | | | | KIR2DL1 |
| --- | --- | --- | --- | --- | --- | --- | --- | --- | --- | --- |
| 0.001 | <0.001 |  | | | | | | | | KIR2DL2 |
| <0.001 | <0.001 | <0.001 |  | | | | | | | KIR2DL3 |
| 0.78 | 0.04 | 0.06 | <0.001 |  | | | | | | KIR3DL1 |
| 0.91 | 0.002 | <0.001 | <0.001 | <0.001 |  | | | | | KIR2DS1 |
| 0.001 | <0.001 | <0.001 | 0.02 | <0.001 | <0.001 |  | | | | KIR2DS2 |
| 0.22 | <0.001 | <0.001 | <0.001 | <0.001 | <0.001 | <0.001 |  | | | KIR2DS3 |
| 0.69 | 0.09 | 0.02 | <0.001 | <0.001 | 0.04 | 0.001 | <0.001 |  | | KIR2DS4 |
| 0.73 | <0.001 | 0.002 | <0.001 | <0.001 | 0.006 | 0.42 | <0.001 | <0.001 |  | KIR2DS5 |
| 0.35 | 0.12 | <0.001 | <0.001 | <0.001 | 0.008 | <0.001 | <0.001 | <0.001 | <0.001 | KIR3DS1 |
| KIR2DL1 | KIR2DL2 | KIR2DL3 | KIR3DL1 | KIR2DS1 | KIR2DS2 | KIR2DS3 | KIR2DS4 | KIR2DS5 | KIR3DS1 |  |

**Figure S1. Linkage between the KIRs in the HTLV-1 (top) and HCV (bottom) cohorts.** Positive linkage disequilibrium (LD) is shown in grey and negative in white. The statistical significance (p-values) of the LD is given in the cells of the tables.

## KIR2DL2 or KIR2DS2?

The above analysis suggests that *KIR2DL2* is most likely to be the primary gene driving the observed effect*. KIR2DL2* is in particularly tight LD with *KIR2DS2*, an activating receptor. It could be argued that an activatory receptor is more likely to modulate CTL and so we specifically investigated whether the observed effect was more likely to be driven by *KIR2DL2* or *KIR2DS2*. In all cases logistic regression models to predict status where HLA allele:*KIR2DL2* and HLA allele:*KIR2DS2* (plus confounders) were included as simultaneous covariates then the covariate HLA:KIR2DL2 was more significant than HLA:KIR2DS2 in every case (i.e. for HLA-*B*57* in HCV, *B*54* in HTLV and *C*08* in HTLV). However, in a model where *HLA:KIR2DS2* but not *HLA:KIR2DL2* was a covariate then HLA:KIR2DS2 was significant for B*54 and B*57 (but not C*08). For *B*54* and *B*57* we therefore also investigated whether the HLA allele was significant in the cohort that was *KIR2DL2*-positive/*KIR2DS2*-negative and vice versa. Cohort sizes were very small but in every case the allele was significant in *KIR2DL2*-positive/*KIR2DS2*-negative but not *KIR2DL2*-negative/*KIR2DS2*-positive (Table S3). The small cohort sizes makes it impossible to conclude that *KIR2DS2* does not also have an effect but it is clear that even in the absence of *KIR2DS2* then *KIR2DL2* does have an effect.

|  | OR (*p value*)  Cohort size | |
| --- | --- | --- |
|  | *KIR2DL2+KIR2DS2-* | *KIR2DL2-KIR2DS2+* |
| HTLV-1, HLA-*B*54* | 18.52 *(p=0.016)*  B*54+=10, N= 48 | 0.007 *(p=1)*  B*54+=2, N= 6 |
| HCV, HLA-*B*57* | 0.47 *(p=0.02)*  B*57+=2, N=13 | 1 *(p=1)*  B*57+=1, N=8 |
| **Table S3. *KIR2DL2* enhancement of the HLA-mediated effect is seen in the absence of KIR2DS2.** We note that the right hand column (KIR2DL2-KIR2DS2+) has been included for completeness but the numbers are far too small to draw any conclusions. | | |

Together these 3 observations indicate that *KIR2DL2* rather than *KIR2DS2* is more likely to be the primary gene driving the enhancement of HLA class I-mediated antiviral immunity.

In summary, the evidence suggests that *KIR2DL2* is most likely to be the KIR which is enhancing immunity. The one KIR for which it is impossible to assess whether it has a stronger effect than *KIR2DL2* is *KIR2DL3* as *KIR2DL2* and *KIR2DL3* segregate as alleles of the same locus. Both are inhibitory but KIR2DL2 provides stronger inhibitory signals than KIR2DL3 [2]. The *2DL2/L3* locus is present in one copy in the majority of haplotypes so the observation that *KIR2DL2* is present in an individual (1 or 2 copies) implies that there are 0 or 1 copies of *KIR2DL3*. So the statement that the presence of *KIR2DL2* enhances class I mediated immunity can be restated in the reciprocal as lack of *KIR2DL3* homozygosity enhances class I mediated immunity. However, functionally it is difficult to understand how the lack of homozygosity for a weaker receptor should enhance immunity more effectively than homozygosity.

## The cumulative presence of stimulatory receptors

*KIR2DL2* is usually present on haplotypes which contain multiple stimulatory KIRs ([3] and section 8 below). We therefore also explored the possibility that the cumulative presence of multiple stimulatory KIR (rather than any one individual KIR) is driving the KIR2DL2 effect. In HCV infection, for each individual, we counted the number of stimulatory receptors of the KIR B haplotype (KIR2DS1, KIR2DS2, KIR2DS3, KIR2DS5, KIR3DS1). Then we simultaneously investigated 1) the effect of B*57 combined with 1 or 2 stimulatory receptors, 2) the effect of B*57 in the presence 3, 4 or 5 stimulatory receptors and 3) the effect of B*57 in the presence of KIR2DL2. Using backward elimination, we found that only the B*57-KIR2DL2 had a significant effect on the outcome of infection. Similar analysis for viral load was not possible because of limited cohort sizes. In HTLV-1 infection, we could apply the same approach only for C*08 and the status variable because of limiting numbers. The stimulatory KIRs available in the cohort were KIR2DS2, KIR2DS3 and KIR3DS1 so we considered individuals with only 1 KIR stimulatory receptor or with 2-3 stimulatory receptors. We found that, as for B*57 in HCV, using backward elimination only C*08-KIR2DL2 had a significant impact on outcome. These two results taken together suggest that KIR2DL2 rather than the cumulative presence of stimulatory receptors of KIR B haplotype enhance the HLA class I-mediated immunity.

# Canonical KIR-HLA binding

## HTLV-1: other group *C1* HLA alleles do not exhibit the same behaviour as *C*08*

We found that, in HTLV-1 infection, *HLA-C*08* was associated with a protective effect: reducing the risk of HAM/TSP and reducing proviral load in ACs. This effect was enhanced in the presence of *KIR2DL2* but reduced/absent in the absence of *KIR2DL2*. We hypothesised that the effect of *KIR2DL2* on the *C*08* protective effect is not mediated via a canonical, direct KIR-HLA interaction. To test this hypothesis we first investigated whether the other group *C1* alleles exhibited the same behaviour as *C*08* in HTLV-1 infection. Virtually all HTLV-1 infected individuals possess at least one *C1* allele (430/432) therefore it was not possible to look at presence or absence of the *C1* ligand instead we looked at the impact of *C1* homozygosity. Grouping all the *C1* alleles we find no significant association between *C1* homozygosity and disease status either in the whole cohort or in the context of *KIR2DL2*. Similarly, *C1/C1* was not associated with decreased proviral load in either ACs or HAM/TSP patients; nor did this become significant in the context of *KIR2DL2*.

In case the failure to find that the *C1* grouping behaved the same as *C*08* was because we were forced to looked at homozygosity for *C1* rather than presence or absence as we had done for *C*08* we additionally, investigated the individual *C1* alleles (see Tables S4 and S5). This confirmed the hypothesis that the protective *C*08* effect and its enhancement by *KIR2DL2* we had observed was not exhibited by other group *C1* alleles.

| HLA-C1 Allele | OR  (whole cohort) | *KIR2DL2*  Genotype | OR  (stratified cohort) | p- value | Allele  Carriers | Cohort size |
| --- | --- | --- | --- | --- | --- | --- |
| *C*01* | 1.302  (p=0.296) | + | 3.257 | 0.028 | 43 | 102 |
|  |  | _ | 0.952 | 0.877 | 129 | 300 |
| *C*03* | 0.594  (p=0.038) | + | 0.751 | 0.561 | 51 | 102 |
|  |  | _ | 0.507 | 0.029 | 138 | 300 |
| *C*07* | 1.213  (p=0.495) | + | 2.985 | 0.070 | 26 | 102 |
|  |  | - | 0.867 | 0.686 | 70 | 300 |
| *C*08* | 0.470  (p=0.032) | + | 0.160 | 0.020 | 14 | 102 |
|  |  | - | 0.665 | 0.364 | 44 | 300 |
| *C*12* | 0.891  (p=0.704) | + | 0.53 | 0.342 | 15 | 102 |
|  |  | - | 1 | 0.993 | 68 | 300 |
| *C*14* | 1.736  (p=0.073) | + | 0.691 | 0.553 | 21 | 102 |
|  |  | - | 2.331 | 0.024 | 66 | 300 |
| **Table S4. Impact of *HLA-C1* alleles on HTLV-1 disease status.** | | | | | | |

| **ACs** | | | | | | |
| --- | --- | --- | --- | --- | --- | --- |
| HLA-C1 Allele | Difference in VL  (whole cohort) | *KIR2DL2*  Genotype | Difference in VL  (stratified cohort) | p- value | Allele  Carriers | Cohort size |
| *C*01* | -0.164  (p=0.234) | + | -0.358 | 0.233 | 15 | 48 |
|  |  | _ | -0.110 | 0.516 | 55 | 132 |
| *C*03* | 0.113  (p=0.407) | + | -0.047 | 0.866 | 25 | 48 |
|  |  | _ | 0.171 | 0.313 | 66 | 132 |
| *C*07* | 0.222  (p=0.187) | + | 0.408 | 0.230 | 10 | 48 |
|  |  | - | 0.146 | 0.490 | 28 | 132 |
| *C*08* | -0.330  (p=0.047) | + | -0.660 | 0.066 | 10 | 48 |
|  |  | - | -0.286 | 0.181 | 26 | 132 |
| *C*12* | 0.070  (p=0.675) | + | -0.134 | 0.716 | 8 | 48 |
|  |  | - | 0.154 | 0.442 | 30 | 132 |
| *C*14* | 0.008  (p=0.964) | + | 0.164 | 0.614 | 11 | 48 |
|  |  | - | -0.164 | 0.455 | 23 | 132 |
| **HAM/TSP** | | | | | | |
| HLA-C1 Allele | Difference in VL  (whole cohort) | *KIR2DL2*  Genotype | Difference in VL  (stratified cohort) | p- value | Allele  Carriers | Cohort size |
| *C*01* | 0.183  (p=0.033) | + | 0.402 | 0.011 | 28 | 54 |
|  |  | _ | 0.143 | 0.152 | 74 | 168 |
| *C*03* | -0.169  (p=0.049) | + | -0.047 | 0.775 | 26 | 54 |
|  |  | _ | -0.220 | 0.028 | 72 | 168 |
| *C*07* | -0.057  (p=0.554) | + | -0.074 | 0.682 | 16 | 54 |
|  |  | - | 0.015 | 0.891 | 42 | 168 |
| *C*08* | -0.173  (p=0.208) | + | -0.856 | 0.005 | 4 | 54 |
|  |  | - | -0.087 | 0.578 | 24 | 168 |
| *C*12* | -0.005  (p=0.965) | + | -0.175 | 0.472 | 7 | 54 |
|  |  | - | -0.004 | 0.973 | 38 | 168 |
| *C*14* | 0.085  (p=0.408) | + | -0.019 | 0.923 | 10 | 54 |
|  |  | - | 0.068 | 0.555 | 43 | 168 |
| **Table S5. Impact of *HLA-C1* alleles on HTLV-1 proviral load** | | | | | | |

## HTLV-1: the *B*54* effect cannot be attributed to linkage with *C*01*

*B*54*, a group *Bw6* HLA allele, is not known to bind any KIR and so we tested whether the observed *B*54* effect was attributable to *C*01* which is in linkage disequilibrium with *B*54* and does encode molecules which bind KIR2DL2. We found that *B*54* rather than *C*01* appeared to be the primary gene associated with HAM/TSP whose detrimental effect was enhanced by *KIR2DL2* (see above section 3.2).

## HCV: the *B*57-KIR2DL2* effect cannot be attributed to *B*57* linkage with HLA molecules that do bind KIR2DL2

*HLA-B*57* is in linkage with 3 HLA class I alleles: *A*01*, *C*18* and *C*06*. None of these HLA molecules is expected to bind KIR2DL2; furthermore *A*01*, *C*0*6 and *C*18* do not have a significant impact on HCV status either overall or in the context of *KIR2DL2*. We therefore conclude that the *B*57-KIR2DL2* effect cannot be attributed to *B*57* linkage with HLA molecules that do bind KIR2DL2.

## HCV: the *B*57-KIR2DL2* effect cannot be attributed to *KIR2DL2* linkage with other KIRs that do bind B*57

HLA-B*57 does not bind KIR2DL2 however B*57 does bind KIR3DL1 and possibly KIR3DS1 both of which are in weak linkage disequilibrium with KIR2DL2 (p=0.04 and p=0.1 respectively). We therefore examined whether the *KIR2DL2* enhancement of the *B*57* protective effect could instead be explained by *KIR3DL1* or *3DS1*. Four observations argue against this:

1. If B*57 binding of KIR3DL1 or 3DS1 was associated with enhanced immunity then other HLA B ligands of KIR3DS1 or KIR3DS1 with similar binding to B*57 would be expected to show a similar pattern to B*57. This was not observed.

| KIR | HLA ligand | OR | p | N KIR+HLA+ | Cohort size |
| --- | --- | --- | --- | --- | --- |
| *3DL1* | Bw4 | 0.94 | 0.7 | 485 | 782 |
| *3DL1* | Bw4.80I | 0.88 | 0.5 | 291 | 782 |
| *3DL1* | Bw4/Bw4 | 1.19 | 0.5 | 131 | 782 |
| *3DL1* | Bw4.80I/Bw4.80I | 1.85 | 0.1 | 47 | 782 |
| *3DS1* | Bw4 | 0.93 | 0.7 | 159 | 782 |
| *3DS1* | Bw4.80I | 0.8 | 0.4 | 91 | 782 |
| *3DS1* | Bw4/Bw4 | 0.94 | 0.9 | 45 | 782 |
| *3DS1* | Bw4.80I/Bw4.80I | 0.61 | 0.4 | 12 | 782 |
| **Table S6. Only the effect of *B*57* and not of alleles with similar binding is enhanced by *KIR2DL2.*** | | | | | |

1. Examining the enhancement of *B*57* by *KIR2DL2*, *KIR3DL1* and *KIR3DS1* it can be seen that the strongest enhancement is by *KIR2DL2.*

| Cohort stratification | OR for *B*57* | P value | N *B*57+* | N *B*57-* |
| --- | --- | --- | --- | --- |
| *KIR2DL2+* | 0.40 | 0.007 | 49 | 359 |
| *KIR2DL2-* | 0.83 | 0.631 | 35 | 339 |
| *KIR3DL1+* | 0.56 | 0.021 | 80 | 658 |
| *KIR3DL1-* | 2.5 | 0.6 | 4 | 40 |
| *KIR3DS1+* | 0.43 | 0.05 | 30 | 214 |
| *KIR3DS1-* | 0.67 | 0.2 | 54 | 484 |
| **Table S7. KIRs which are known to bind B*57 do not enhance the *B*57* protective effect as much as KIR2DL2 does.** | | | | |

1. If we exclude *KIR2DL2+* individuals then neither *KIR3DL1* nor *KIR3DS1* enhance *B*57* (in *KIR2DL2-3DL1+* OR =0.83 p= 0.63. *KIR2DL2-3DS1+* OR =0.98 p= 0.97). However there are only 11 *KIR2DL2-3DS1+ B*57+* individuals (35 KIR2DL2-3DL1+ B*57+) so loss of significance in the latter case may be attributable to power. Conversely, if we exclude *KIR3DS1+* individuals then there is still a trend for *KIR2DL2* to enhance *B*57* (OR=0.47 p=0.092) even though there are only 30 *KIR2DL2+KIR3DS1-* individuals with *B*57*. There are only 4 individuals who are *KIR2DL2+* but *KIR3DL1-* so excluding *KIR3DL1* individuals is not possible.
2. In a model to predict HCV status in which *KIR2DL2* with *B*57* and *KIR3DL1* with *B*57* were both included as covariates (plus confounders) and then non significant HLA:KIR were removed by backwards stepwise exclusion then only *KIR2DL2* with *B*57* remained as a significant predictor. Similarly, in a model to predict HCV status in which *KIR2DL2* with *B*57* and *KIR3DS1* with *B*57* were both included as covariates (plus confounders) then, following backwards stepwise exclusion, only *KIR2DL2* with *B*57* remained as a significant predictor.

# Does the effect of KIR2DL2 depend on the presence of its ligands?

KIR2DL2 binds HLA group C1 molecules and, with weaker affinity, C2 molecules [2]. We hypothesised that KIR2DL2-dependent enhancement of HLA-mediated immunity would be greatest in individuals bearing one or two copies of the group *C1* ligand. This required further stratification of the cohorts and only the size of the HCV cohort allowed for such calculations. We indeed found that in *KIR2DL2+* individuals who had at least one *C1* ligand, the effect of *B*57* was protective (OR=0.34, p=0.008, B57+=31, N=332) and it was slightly weakened when *KIR2DL2* was present without its *C1* ligand (OR=0.4, p=0.38, B57+=18, N=76). However, the differences are not large, possibly because KIR2DL2 also binds C2 molecules and so there are no people in which KIR2DL2 does not have a ligand.

# The cumulative presence of inhibitory KIRs

We quantified the magnitude of KIR inhibitory signals per individual by counting the number of inhibitory KIR that were present with their ligand (KIR2DL1:C2, KIR2DL2:C1, KIR2DL3:C1, KIR3DL1:Bw4I [4-6]). Then, we stratified the cohort for “low inhibitory signal”, count ≤1, and “high inhibitory signal”, count ≥2. The cut-off (count ≤1, count ≥2) was chosen so that there were similar numbers of individuals in the two strata. In HCV infection, we found that *B*57* had an increased protective effect for individuals within the “high inhibitory signal” category (OR=0.48, p=0.03, B57+=50, N=518) but the effect was not significant for “low inhibitory signal” individuals (OR=0.65, p=0.3, B57+=34, N=262). In HTLV-1 infection, the protective effect of *C*08* was pronounced among individuals with a “high inhibitory signal” (OR=0.30, p=0.02, C*08+=29, N=242) but absent in the “low inhibitory signal” group (OR=0.98, p=0.97, C*08+=29, N=160). Similarly, the detrimental impact of *B*54* was increased in individuals that possessed more inhibitory KIRs with their ligands (OR= 5.31, p=0.001, B54+=43, N=242) compared to individuals with a “low inhibitory signal” score (OR= 1.58, p=0.36, B54+=42, N=160). However, the effect of the “high inhibitory KIR signal” could be attributable to *KIR2DL2* as the “high inhibitory signal” group is heavily enriched for individuals with *KIR2DL2* (especially for the HTLV-1 cohort). Additionally, the observation (section 8 below) that the KIR haplotype *AA* does not have an enhancing effect suggests that even if other inhibitory signals may contribute, *KIR2DL2* is necessary for a detectable effect.

# The role of KIR haplotypes

Two broad groups of KIR haplotypes have been defined: A haplotypes and B haplotypes [3]. A haplotypes are dominated by inhibitory KIRs (having 2 activating and 5 inhibitory KIRs); B haplotypes which are less tightly defined, are considered more activatory (having up to 7 activating KIRs and on average 5 inhibitory KIRs). *A* haplotypes do not have *KIR2DL2*, *B* haplotypes can but do not always include *KIR2DL2*. We investigated whether different haplotypes were associated with a different enhancement of the HLA class I associations.

For each individual, the following rules for attributing a KIR haplotype were used. *AA*: only *KIR2DL1, 3DL1, 2DL3 and 2DS4* are present; *AB*: all the KIRs in haplotype *A* were present as well as one or more of *KIR2DL2, 2DS2, 2DS3, 2DS5* or *3DS1* and *BB*: not all the KIRs in haplotype *A* were present and at least one of the KIRs in haplotype *B* were present.

We found (Table S8) that the *AA* haplotypes did not have an effect either on outcome of infection, nor on viral burden for any of the three HLA molecules studied. The *AB* KIR haplotypes enhanced the effect of both protective and detrimental molecules, for all three HLA molecules studied. The fact that AB but not AA enhances HLA associations suggests that it is the B haplotype which is responsible for the AB enhancement. Unfortunately, there are insufficient numbers of individuals with *BB* to draw any conclusions about *BB*.

|  | **KIR Haplotype** | | |
| --- | --- | --- | --- |
|  | ***AA*** | ***AB*** | ***BB*** |
|  | **OR**  (p-value, allele carriers, cohort size) | | |
| HCV B*57 status | 0.73  (p=0.5, n=22, N=252) | 0.57  (p=0.1, n=44, N=405) | 0.36  (p=0.07, n=18, N=125) |
| HTLV-1 C*08 status | 0.70  (p=0.54, n=28, N=202) | 0.38  (p=0.07, n=30, N=190) | _ |
| HTLV-1 B*54 status | 1.23  (p=0.65, n=46, N=202) | 5.35  (p=0.003, n=37, N=190) | _ |
| **Table S8. The role of *KIR* haplotypes** | | | |

To investigate the enhancement afforded by the B haplotype we split the B+ individuals into people with and without *KIR2DL2* (Table S9). This clearly showed that the B-enhancement was absent when *KIR2DL2* was absent and present when *KIR2DL2* was present. We conclude that a haplotype analysis offers little additional information, *KIR-A* and *KIR-B* simply being imperfect markers for absence or presence of *KIR2DL2*.

|  | **KIR Haplotype** | | |
| --- | --- | --- | --- |
|  | ***B+(AB or BB)*** | ***B+KIR2DL2+*** | ***B+KIR2DL2-*** |
|  | **OR**  (p-value, allele carriers, cohort size) | | |
| HCV B*57 status | 0.51  (p=0.02, n=62, N=530) | 0.40  (p=*0.007*, n=49, N=408) | 1.23  (p=0.75, n=13, N=122) |
| HTLV-1 C*08 status | 0.35  (p=0.053, n=30, N=200) | 0.16  (p=0.02, n=14, N=102) | 0.69  (p=0.64, n=16, N=98) |
| HTLV-1 B*54 status | 6.25  (p=0.001, n=39, N=200) | 12.50  (p=0.004, n=21, N=102) | 2.78  (p=0.18, n=18, N=98) |
| **Table S9. *KIR-B* enhancement is not seen in the absence of *KIR2DL2*** | | | |

# A*02 binds peptides strongly

We tested the hypothesis that A*02 molecules binds peptides significantly more strongly than other HLA molecules using two approaches. First, we extracted the list of positive MHC Binding Assays from the Immune Epitope Database (IEDB) [7] which contained in total 78,202 peptides that bind HLA molecules. We then compared the experimental affinity measurements of all the A*02xx molecules with the corresponding values of other frequent HLA molecules (only HLA-A and B molecules were considered). The frequencies of the HLA molecules were calculated based on available data for UK and USA populations at The Allele Frequency Net Database [8]. Secondly, we used the epitope prediction software Metaserver [9] to identify potential epitopes for both the HCV and HTLV-1 proteomes for 36 and 21 HLA molecules respectively (we only obtained predicted epitopes for the HLA alleles present in the cohorts included in the study). Then we compared the predicted affinity of the A*02xx:peptide complexes with the predicted values for the other pMHC complexes for all peptides which were predicted to bind (affinity less than 500 nM IC50). Both approaches show that A*02 binds peptides significantly more strongly compared to other HLA molecules. In the first approach (experimentally measured affinities), A*02 bound epitopes significantly more strongly than each of A*01, A*30, B*07, B*35, B*44 and B*45 (p<0.00001 in each case, Wilcoxon Rank sum, Figure S2). In the second approach (theoretically predicted affinitites), A*02 bound peptides significantly more strongly than the other A alleles considered (HTLV-1: p=0.015, HCV: p<0.00001), B alleles (HTLV-1: p<0.00001, HCV p<0.00001) and A and B alleles combined (HTLV-1: p<0.00001, HCV p<0.00001), data not shown.

**
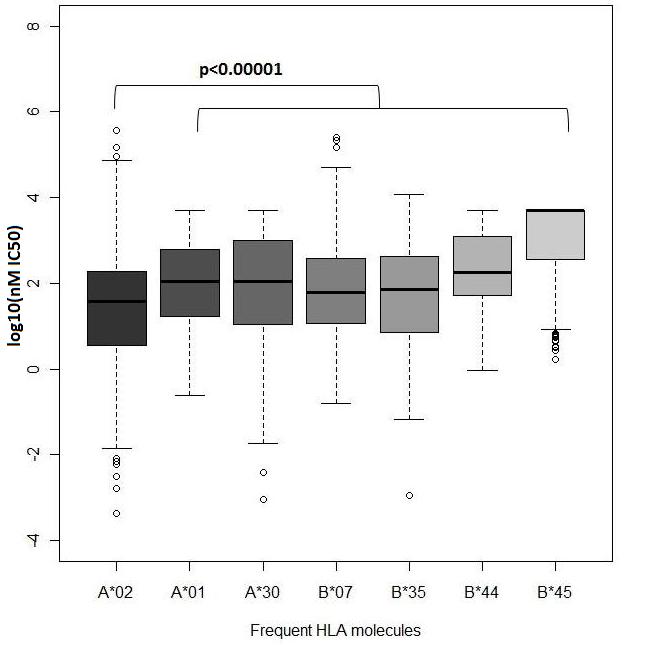
**

**Figure S2. *A*02* binds peptides significantly more strongly that other HLA-molecules which are frequent in UK and USA populations.** The binding measure shown here is the affinity (nM IC50) values obtained from the IEDB database for 16,958 peptides.
